# Non-Invasive Ventilation Applied for Recovery from Exercise-Induced Diaphragmatic Fatigue

**DOI:** 10.2174/1874306400802010016

**Published:** 2008-02-26

**Authors:** Hans-Joachim Kabitz, David Walker, Stephan Prettin, Stephan Walterspacher, Florian Sonntag, Michael Dreher, Wolfram Windisch

**Affiliations:** a Department of Pneumologya, University Hospital Freiburg, Killianstrasse 5, D-79106 Freiburg, Germany; b Department of Sports-Medicine, University Hospital Freiburg, Killianstrasse 5, D-79106 Freiburg, Germany

**Keywords:** Inspiratory muscles, twitch pressures, exercise testing, ergospirometry, respiratory physiology.

## Abstract

**Background:**

Exercise-induced diaphragmatic fatigue (DF) is conventionally considered to reflect impaired diaphragm function resulting from load imposed on the diaphragm during exercise and is known to be reduced by the application of non-invasive ventilation (NIV) during heavy-intensity exercise testing (HEET). On that physiological condition NIV applied for diaphragm unloading during recovery from exercise should be capable of accelerating recovery from DF and therewith prolonging exercise time to exhaustion and limiting the development of DF during a subsequent HEET compared to recovery during spontaneous breathing.

**Methods:**

Seven highly-trained subjects (V’O_2max_ 62.7±7.8 ml/kg/min) performed four HEET at 85% V’O_2max_ with 60 min of recovery during I spontaneous breathing and II NIV between two HEET.

**Results:**

Twitch transdiaphragmatic pressure (TwPdi) during supramaximal magnetic phrenic nerve stimulation decreased (p<0.04) following first HEET and subsequently completely recovered (p>0.2) during I and II. Following second HEET TwPdi comparably decreased (I 0.24±0.21 *vs* II 0.32±0.29 kPa; p=0.17). Exercise time to exhaustion during second HEET was equal during I and II (I 514±49 *vs* II 511±92 s; p=0.88).

**Conclusions:**

In conclusion, NIV applied for diaphragm unloading during recovery following HEET does neither affect recovery from DF nor subsequent exercise performance thereby providing further evidence that DF might reflect post-exercise diaphragm shielding rather than impaired diaphragm function.

## INTRODUCTION

Heavy-intensity exercise testing (HEET) causes fatigue of the human diaphragm [1-3]. This exercise-induced diaphragmatic fatigue is considered to represent an important factor limiting maximal ventilation and exercise performance [4-8]. According to the conventional understanding exercise-induced diaphragmatic fatigue represents impaired diaphragm function resulting from load imposed on the exercising diaphragm [4,6,8]. However, there is rising evidence that exercise-induced diaphragmatic fatigue might serve as means of shielding the human diaphragm during the post-exercise period rather than representing impaired diaphragm function [9,10]. Interestingly, the use of non-invasive ventilation (NIV) applied for inspiratory muscle unloading during HEET has recently been shown to limit the development of exercise-induced diaphragmatic fatigue and to prolong exercise time to exhaustion [11]. However, when NIV is used during recovery from HEET its physiological effects on diaphragmatic force generation and subsequent exercise performance remain unclear. For this reason the current study aimed at assessing physiological effects of NIV applied for inspiratory muscle unloading during the recovery process after HEET. According to the conventional understanding of exercise-induced diaphragmatic fatigue NIV should be capable of accelerating the recovery process from diaphragmatic fatigue and therewith prolonging exercise time to exhaustion and limiting the development of diaphragmatic fatigue during a subsequent HEET when compared to recovery during spontaneous breathing.

## METHODS

All procedures were approved by the Institutional Review Board for Human Studies at the Albert-Ludwigs University Freiburg, Germany. All subjects gave their written informed consent.

### Participants

Seven highly-trained, healthy male amateur cyclists who were not taking any medication were studied. Participants were required to restrict their caffeine-intake and avoid stressful physical activity 24 hours prior to each study phase. All participants had their last meal at least two hours preceding measurements. All measurements were performed at the same time of the day for a particular participant and Study Phase 1 and 2 were separated by 48 hours.

### Lung function, Pressure and Airflow Recordings

Lung function parameters were measured using body-plethysmography (Masterlab-Compact^®^, Jaeger, Hochberg, Germany) according to the ERS statement [12]. All airflow and pressure recordings were measured using a pneumotachograph (ZAN 100 Flowhandy II^®^, ZAN, Oberthulba, Germany) and a pressure transducer (ZAN 400^®^, ZAN, Oberthulba, Germany). PImax sustained for one second (PImax_1.0_) was assessed at residual volume (RV) as previously reported [13]. Twitch mouth (TwPmo), esophageal (TwPes) and gastric (TwPga) pressures were recorded during supramaximal bilateral anterior magnetic phrenic nerve stimulation (BAMPS, Magstim 200^2®^, Magstim, Wales, United Kingdom) [14,15]. Several previous studies have demonstrated that BAMPS reliably achieves supramaximal phrenic nerve stimulation and this was therefore not re-tested in the current protocol [14,16,17]. In addition, BAMPS is known to reliably detect diaphragmatic fatigue [16].

A fully automated and controlled inspiratory pressure trigger was used at 0.5 kPa for TwPmo to initialize recordings of twitch pressures close to functional residual capacity as has been described previously [18,19]. TwPes and TwPga were measured using a thin conventional double-balloon catheter (ZAN, Oberthulba, Germany), placed in the stomach and esophagus (balloons containing 3 and 1.5 ml of air, respectively) based on previous recommendations [20,21]. Twitch transdiaphragmatic pressure (TwPdi), which is currently considered to be the most objective measure of diaphragmatic function and fatigue [22] was calculated by online point-to-point subtraction of TwPes from TwPga. The effects of twitch potentiation [23] following exercise have to be taken into account. For this reason, initially assessed twitch pressures (TwP) at rest on each study phase were applied at a fully potentiated level after performing maximal static inspiratory efforts. Breathing frequency and functional residual capacity as averaged from the last three breaths were automatically calculated and controlled by the computer device [9,18,19].

### Non-Invasive Ventilation

Non-invasive pressure supported ventilation (NIV) was applied (Legendair^®^, Airox, France) in order to achieve maximal respiratory muscle unloading. Inspiratory trigger sensitivity of the ventilator can be set at an arbitrary scale between 1 (high) and 5 (low). A value of 3 was chosen in all participants. Expiration was possible when the maximal inspiratory flow decreased to 75%, according to an expiratory trigger threshold of minus 25%. Inspiratory positive airway pressure was set at individually tolerated maximal level (range 12-15 mbar) and all participants were instructed to relax their respiratory muscles as much as possible in order to allow maximal respiratory muscle unloading. Expiratory positive airway pressure was set to 0 mbar in all subjects. A one-way circuit with an expiratory valve connected to a medium-sized full-face mask (Ultra mirage™, Resmed, Moenchengladbach, Germany) was used. At the end of Study Phase 1 each participant underwent a thorough NIV familiarization process and NIV was well tolerated in all participants.

### Experimental Study Design (Fig. 1)

A computer-controlled, eddy-current-braked cycle ergometer (Lode Excalibur^®^, Groningen, Netherlands) was used for exercise testing. A ramped short-term test beginning at 150 W followed by an increase in work rate of 10 W every 10 seconds until exhaustion [24] was applied to determine maximal oxygen uptake (V’O_2max_) using an ergospirometric device (ZAN 600^®^, ZAN, Oberthulba, Germany) for breath-by-breath recordings of gas exchange and ventilation. From these data individual workloads were deduced to reflect a workload of at least 85% V’O_2max_, considering previous recommendations [25]. The best-tolerated pedaling frequency for each participant was established during the incremental short-term test and subsequently maintained throughout all exercise protocols.

Two consecutive HEET per day were conducted on two different study phases. After a three minute warm-up cycle at 150 W subjects were brought up in 50 W increments per minute to the predefined individual workload requiring at least 85% V’O_2max_ on both study phases. This workload was maintained until volitional exhaustion. Stop criterion for HEET was defined as a pedaling rate falling below 60 revolutions per minute for three consecutive seconds. Subsequently, participants recovered by resting in a supine position for a 60 minute period. During this period, participants recovered by breathing room air on Study Phase 1 while receiving NIV on Study Phase 2.

Following the recovery period, HEET was re-performed until volitional exhaustion using the identical protocols. The duration for the initial HEET on Study Phase 2 was set as isotime to the initial HEET on Study Phase 1. In contrast, the second HEET on Study Phase 1 and 2 was performed until volitional exhaustion as outlined above. Therefore, all procedures were identical for the two study phases, with the exception of the application of NIV.

**Fig. (1).**
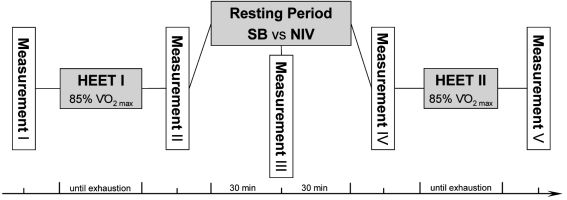
Study protocol. HEET = heavy-intensity exercise testing; NIV = non-invasive ventilation; SB = spontaneous breathing; V’O_2max_ = maximal oxygen uptake.

The following parameters were measured in the same order on both study phases: blood lactate and blood gas values from the arterialized ear lobe, heart rate, PImax_1.0_, twitch pressures and cycling time to exhaustion. A synopsis of the experimental study design is given in Fig. (**1**). These measurements were conducted five times on each study phase (labeled I to V): before and immediately after each HEET as well as during the recovery period (Fig. **1**).

### Statistical Analysis

Statistical analysis was performed using Sigma-Stat^® ^(Systat Software, Inc., Point Richmond, California, USA). Unless otherwise stated, data are presented as mean and standard deviation (SD). Two groups were compared using the paired *t*-test. When comparing more than two groups, one-way analysis of variance with repeated measures (RM-ANOVA) was performed including an all pair-wise comparison using the Holm-Sidak method. For correlation analysis the Pearson Product Moment Correlation was used for metric data. A p-value <0.05 was considered statistically significant.

## RESULTS

Anthropometric data, lung function parameters, and results from the incremental short-term test are presented in Table **1**. Values for blood lactate, blood gas analysis, heart rate, tests on inspiratory muscle strength and cycling time to exhaustion on the two study phases are given in Tables **2** and **3**. TwP were successfully assessed in all but one subject in whom TwPga and hence TwPdi was not available for Study Phase 1 due to technical problems caused by gastric catheter displacement during HEET as described recently [26].

### Study Phase 1 (Spontaneous Breathing)

Exercise time to exhaustion averaged 531±48 s for the first and 514±49 s for the second HEET (p=0.09). Following RM-ANOVA no difference could be detected for PImax values at any time of data collection (p=0.24). TwPmo and TwPes were markedly lower after the first HEET compared to pre-exercise values (p=0.005 and p=0.006, respectively). Values for TwPmo and TwPes did not reach significant differences comparing values prior to and after the second HEET (p=0.26 and p=0.51, respectively). TwPdi significantly decreased following exercise in both HEET (p<0.04 in both instances). Reduction in TwPdi following the first HEET fully recovered after 35 min of recovery when comparing measurement III and I (p=0.2).

### Study Phase 2 (NIV)

Exercise time to exhaustion averaged 524±96 s for the first HEET which was comparable to the second HEET (511±92 s, p=0.3). RM-ANOVA revealed comparable values for all PImax measurements (p=0.31). TwPmo and TwPes values tended to be lower after the first and second HEET, compared to initial values at rest. However, this did not reach statistical significance (p>0.08 in all instances). TwPdi significantly decreased following exercise in both HEET (p<0.03 in both instances). Reduced TwPdi values following the first HEET fully recovered after 35 min of NIV assisted breathing when comparing measurement III and I (p=0.3).

### Comparisons Between Study Phase 1 (Spontaneous Breathing) and Study Phase 2 (NIV)

Exercise time to exhaustion for the first HEET was set to be isotime at Study Phase 1 and 2 (p=0.8). Following RM-ANOVA no significant differences could be detected for any PImax value (p=0.23) when comparing all measurements (I to V) from Study Phase 1 and 2. Values for TwPdi in measurement III, IV and V showed no significant difference when comparing Study Phase 1 and 2 (p>0.1 in all instances). Furthermore, exercise time to exhaustion in the second HEET was not affected by application of NIV (p=0.88). Therefore, NIV did not affect inspiratory muscle strength following exertion and, in particular, did not prolong exercise time to exhaustion. However, heart rate was significantly lower during the resting period when NIV was compared to spontaneous breathing (65±9 versus 76±13 min^-1^, p=0.02). Progression for mean TwPmo and TwPdi for the two study phases are illustrated in Figs. (**2**,**3**), respectively.

## DISCUSSION

The present study aimed at evaluating the use of NIV applied for inspiratory muscle unloading during recovery from HEET and to determine whether its application alters the development of exercise-induced diaphragmatic fatigue or exercise time to exhaustion in a second consecutive HEET. The main finding is that NIV used for inspiratory muscle unloading during recovery from HEET did neither affect recovery from exercise-induced diaphragmatic fatigue nor subsequent exercise performance since both cycling time to exhaustion and diaphragmatic force generation remained unchanged when NIV was used for inspiratory muscle unloading during recovery, rather than spontaneous breathing alone.

These findings are unexpected with regard to the conventional understanding of exercise-induced diaphragmatic fatigue considered to represent impaired diaphragm function resulting from load imposed on the diaphragm during exercise [4,6,8]. In addition, previous work has shown that NIV is capable of reducing respiratory and limb discomfort and of prolonging exercise time to exhaustion when used to unload respiratory muscles during HEET [11, 25]. On the basis of exercise-induced diaphragmatic fatigue reflecting impaired post-exercise diaphragm function [4,6,8] one would expect that respiratory muscle unloading by the use of NIV should be capable of accelerating the recovery process of the impaired diaphragm and of improving subsequent exercise performance by providing more power-reserve for the pre-fatigued inspiratory muscles during subsequent exercise. Since both has shown not to be achievable by the use of NIV applied for respiratory muscle unloading during recovery from HEET these data question whether the underlying physiological mechanisms of diaphragmatic force generation during exercise and exercise-induced diaphragmatic fatigue might differ from the conventionally proposed. It has been shown recently that diaphragmatic force generation progressively increases with increasing workload and that exercise-induced diaphragmatic fatigue manifests *after *– rather than *during* – exercise [9]. In addition, diaphragmatic force generation has been shown to be subject to similar regulations during either whole-body exercise or controlled hyperventilation but to differ markedly during recovery whereas only whole-body exercise induced diaphragmatic fatigue despite equal amounts of load imposed on the inspiratory muscles during the two protocols [10]. These findings provide rising evidence that exercise-induced diaphragmatic fatigue ought to be attributed to non-ventilatory controlled feedback mechanisms and might serve as means of shielding the human diaphragm during the post-exercise period rather than representing impaired diaphragm function [9,10]. In this context the findings of the current study turn out to be physiologically reasonable. First, NIV applied for respiratory muscle unloading during recovery is not capable of accelerating the recovery process from exercise-induced diaphragmatic fatigue since there is no impaired diaphragm function to be restored. Second, the application of NIV during the recovery process does not affect subsequent exercise performance or development of subsequent exercise-induced diaphragmatic fatigue since diaphragm function is not impaired following a preceding HEET.

Interestingly, diaphragmatic fatigue was not consolidated following the second HEET, compared to the first HEET in the current study. In contrast, the opposite was observed in another study, whereby diaphragmatic fatigue was more evident following a second HEET [27]. The reason for this discrepancy remains unclear, but might be explained by different inspiratory muscle fatiguing tasks of the studies. In regard to this, resistive loaded breathing until exhaustion was used in the study by Rohrbach and co-workers, but heavy-intensity whole body exercise was performed in the present study. Therefore, different types of fatiguing tasks to the inspiratory muscles are suggested to induce different regulations of diaphragmatic force generation. However, this issue was not addressed in the current study and needs further investigation.

Both volitional and non-volitional tests on inspiratory muscle strength have been applied in the present study. Diaphragmatic fatigue, as assessed by TwPdi, is known to develop following resistive loaded breathing [28], whereas no fatigue was observed when targeting mouth pressure [29]. This indicates that assessment of different TwP might lead to different results. Therefore, all different TwP were assessed in the present study. Interestingly, global inspiratory muscle strength, as volitionally assessed by PImax, remained unchanged during the whole study. This is in line with previous findings [22,30]. Additionally, with exception of the first HEET during Study Phase 1 TwPmo and TwPes revealed only trends towards lower values after the HEET without reaching statistical significance on the two study phases. In contrast, TwPdi was reduced following both HEET during the two study phases. These results suggest that parts of diaphragmatic force generation are taken over by other inspiratory muscles during prolonged HEET as shown previously [7,8,28]. Here, rib cage muscles play a key role during exercise in pressure generation resulting in a rib cage displacement [31] enabling the diaphragm to increasingly act as a flow rather than a pressure generator [32].

There are certain limitations of the current study which need to be addressed. The sample size was small due to the complex and demanding study design. For this reason the present findings might not be generalized and further studies are needed to verify these data. However, the current sample size was comparable to previous studies dealing with the same field of research [11,27,28,30] outlining the difficulties between practical feasibility and statistical ideals in this area of research. Next, the progression of diaphragmatic force generation could have been more precisely assessed by performing additional measurements during the recovery phase. However, this would have resulted in insufficient NIV during the recovery process and was therefore omitted. Finally, the results of the present study are restricted to highly-trained subjects only, and further studies are needed in additional cohorts.

In conclusion, the present study applied NIV for inspiratory muscle unloading during recovery following HEET prior to subsequent HEET. Here NIV did neither affect recovery from exercise-induced diaphragmatic fatigue nor subsequent exercise performance since both cycling time to exhaustion and diaphragmatic force generation remained unchanged when NIV was used for inspiratory muscle unloading during recovery, rather than spontaneous breathing alone. These findings provide further evidence that exercise-induced diaphragmatic fatigue might reflect post-exercise diaphragm shielding rather than impaired diaphragm function.

## Figures and Tables

**Fig. (2).**
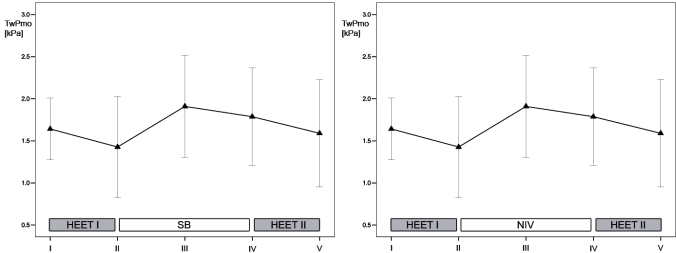
Progression of twitch mouth pressures (TwPmo) for the two consecutive exercise protocols and recovery phase during spontaneous breathing (left panel) and during non-invasive ventilation (right panel). Data are presented as mean. T-bars represent 95% confidence interval of the mean. I-V represent consecutive measurements. HEET = heavy-intensity exercise testing; NIV = non-invasive ventilation; SB = spontaneous breathing.

**Fig. (3).**
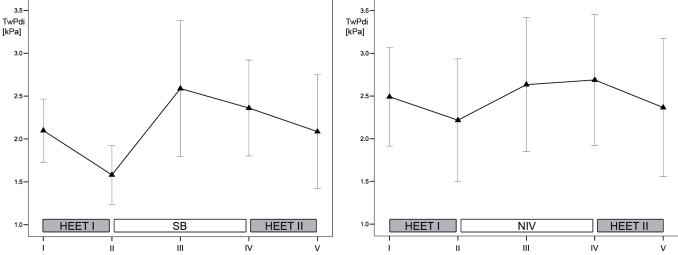
Progression of twitch transdiaphragmatic pressures (TwPdi) for the two consecutive exercise protocols and recovery phase during spontaneous breathing (left panel) and during non-invasive ventilation (right panel). Data are presented as mean. T-bars represent 95% confidence interval of the mean. I-V represent consecutive measurements. HEET = heavy-intensity exercise testing; NIV = non-invasive ventilation; SB = spontaneous breathing.

**Table 1.** Anthropometric Data, Lung Function and Performance Parameters

	Age	Height	BMI	FVC	FEV_1_	RV	V’O_2max_	WL_max_	HR_max_
	[a]	[cm]	[kg/m^2^]	[% pred]	[% pred]	[% pred]	[ml/kg/min]	[W]	[min^-1^]
Mean	27.7	181	21.2	112	108	99	62.7	466	189
SD	3.8	4	1.1	15	10	9	7.8	45	10

BMI = body mass index; FEV_1_ = forced expiratory volume in 1 second; FVC = forced vital capacity; HR_max_ = maximal heart rate; RV = residual volume; V’O_2max_ = maximal oxygen uptake; WL_max_ = maximal workload.

**Table 2.** Exercise Parameters and Tests on Inspiratory Muscle Strength on Study Phase 1

		HEET 1		SB	HEET 2	
		I	II	III	IV	V
**TwPmo**	[kPa]	1.59 ± 0.31	1.04 ± 0.23	1.84 ± 0.65	1.66 ± 0.43	1.46 ± 0.57
**TwPes **	[kPa]	1.38 ± 0.29	0.92 ± 0.19	1.58 ± 0.51	1.50 ± 0.41	1.37 ± 0.48
**TwPga **	[kPa]	-0.78 ± 0.30*	-0.76 ± 0.23*	-1.09 ± 0.49*	-0.97 ± 0.34*	-0.76 ± 0.39*
**TwPdi **	[kPa]	2.10 ± 0.35*	1.58 ± 0.33*	2.59 ± 0.76*	2.36 ± 0.53*	2.09 ± 0.63*
**PImax **	[kPa]	13.1 ± 1.5	13.2 ± 1.6	12.9 ± 1.4	12.4 ± 0.9	12.6 ± 1.7
**HR**	[min^-1^]	71 ± 8	185 ± 10	76 ± 13	75 ± 11	185 ± 8
**BL**	[mg/dl]	1.7 ± 0.5	12.2 ± 1.6	3.9 ± 1.3	2.0 ± 0.5	11.0 ± 2.3
**PaO_2_**	[mmHg]	88.3 ± 7.2	104.3 ± 10.2	85.8 ± 5.8	83.5 ± 5.3	101.6 ± 8.4
** PaCO_2_**	[mmHg]	36.0 ± 2.6	31.4 ± 1.6	31.9 ± 5.8	34.8 ± 2.9	31.2 ± 3.3
**pH**		7.41 ± 0.03	7.20 ± 0.06	7.41 ± 0.06	7.41 ± 0.02	7.22 ± 0.08

BL = blood lactate; HEET = heavy-intensity exercise testing; HR = heart rate; PaCO_2_ = partial pressure of carbon dioxide; PaO_2_ = partial pressure of oxygen; PImax = maximal inspiratory pressure; SB = spontaneous breathing; TwPdi = twitch transdiaphragmatic pressure; TwPes = twitch esophageal pressure; TwPga = twitch gastric pressure; TwPmo = twitch mouth pressure.

* n=6.

**Table 3.** Exercise Parameters and Tests on Inspiratory Muscle Strength on Study Phase 2

		HEET 1		NIV	HEET 2	
		I	II	III	IV	V
**TwPmo**	[kPa]	1.64 ± 0.40	1.43 ± 0.65	1.91 ± 0.65	1.79 ± 0.63	1.59 ± 0.68
**TwPes**	[kPa]	1.43 ± 0.42	1.24 ± 0.60	1.63 ± 0.60	1.63 ± 0.61	1.45 ± 0.60
**TwPga**	[kPa]	-1.20 ± 0.43	-1.10 ± 0.38	-1.14 ± 0.34	-1.16 ± 0.36	-1.01 ± 0.40
**TwPdi**	[kPa]	2.49 ± 0.62	2.22 ± 0.78	2.63 ± 0.85	2.69 ± 0.83	2.37 ± 0.87
**PImax**	[kPa]	12.0 ± 1.9	12.5 ± 1.7	12.2 ± 1.4	12.1 ± 1.7	12.5 ± 1.1
**HR**	[min^-1^]	71 ± 12	184 ± 9	65 ± 9	67 ± 8	187 ± 9
**BL**	[mg/dl]	1.6 ± 0.4	11.2 ± 1.6	3.7 ± 1.5	2.3 ± 0.7	9.8 ± 2.2
**PaO_2_**	[mmHg]	82.9 ± 7.4	99.9 ± 14.5	86.3 ± 17.7	83.0 ± 14.0	89.3 ± 12.0
**PaCO_2_**	[mmHg]	37.8 ± 3.2	31.1 ± 4.4	30.6 ± 7.4	30.1 ± 6.9	30.1 ± 2.3
**pH**		7.41 ± 0.02	7.23 ± 0.03	7.45 ± 0.06	7.49 ± 0.07	7.25 ± 0.05

BL = blood lactate; HEET = heavy-intensity exercise testing; HR = heart rate; NIV = non-invasive ventilation; PaCO_2_ = partial pressure of carbon dioxide; PaO_2_ = partial pressure of oxygen; PImax = maximal inspiratory pressure; TwPdi = twitch transdiaphragmatic pressure; TwPes = twitch esophageal pressure; TwPga = twitch gastric pressure; TwPmo = twitch mouth pressure.
